# An Elegant Analysis of *White Spot Syndrome Virus* Using a Graphene Oxide/Methylene Blue based Electrochemical Immunosensor Platform

**DOI:** 10.1038/srep46169

**Published:** 2017-04-10

**Authors:** Anusha Natarajan, K. S. Shalini Devi, Sudhakaran Raja, Annamalai Senthil Kumar

**Affiliations:** 1 Aquaculture Biotechnology Laboratory, Department of Integrative Biology, School of Biosciences and Technology, Vellore Institute of Technology University, Vellore-632014, Tamil Nadu, India; 2 Nano and Bioelectrochemistry Research Laboratory, Department of Chemistry, School of Advanced Sciences, Vellore Institute of Technology University, Vellore-632014, Tamil Nadu, India; 3 Carbon dioxide Research and Green Technology Centre, Vellore Institute of Technology University, Vellore 632014, Tamil Nadu, India

## Abstract

White spot syndrome virus (WSSV) is a major devastating virus in aquaculture industry. A sensitive and selective diagnostic method for WSSV is a pressing need for the early detection and protection of the aquaculture farms. Herein, we first report, a simple electrochemical immunosensor based on methylene blue dye (MB) immobilized graphene oxide modified glassy carbon electrode (GCE/GO@MB) for selective, quick (35 ± 5 mins) and raw sample analysis of WSSV. The immunosensor was prepared by sequential modification of primary antibody, blocking agent (bovine serum album), antigen (as vp28 protein), secondary antibody coupled with horseradish peroxidase (Ab2-HRP) on the GCE/GO@MB. The modified electrode showed a well-defined redox peak at an equilibrium potential (E_1/2_), −0.4 V vs Ag/AgCl and mediated H_2_O_2_ reduction reaction without any false positive result and dissolved oxygen interferences in pH 7 phosphate buffer solution. Under an optimal condition, constructed calibration plot was linear in a range of 1.36 × 10^−3^ to 1.36 × 10^7^ copies μL^−1^ of vp28. It is about four orders higher sensitive than that of the values observed with polymerase chain reaction (PCR) and western blot based WSSV detection techniques. Direct electrochemical immunosensing of WSSV in raw tissue samples were successfully demonstrated as a real sample system.

White spot syndrome virus (WSSV), a new family of viruses named Nimaviridae, genus Whispovirus^1^, is a highly lethal, contagious and the most serious viral pathogen to *penaeid* shrimp. It causes 100% mortality within 3–7 days of attack^2^. Since the first outbreak in Taiwan in 1992^3^, WSSV has been spreading worldwide and resulting in huge economic loss in the shrimp aquaculture industries^4^. In India, the gross economic loss due the WSSV attack was estimated as 48717 metric-ton of shrimp, which is equivalent to USD 150 million and employment of 2.15 million man days^5^. WSSV disseminates quickly under normal environmental condition and infects a wide host range of host which includes copepods, crab, lobster, cray-fish and prawn. Forty structural proteins of WSSV have been discovered till now. Amongst them, vp28 protein plays a major role in binding and penetration of virus in the host cell^1^. *Paratelphusa hydrodomous*, a highly-susceptible rice water crab for WSSV viral infection, has been widely used as a model organism for the pathological investigation^6^. For the first time in this work, we report a simple and selective electrochemical immunosensor for a quick (35 ± 5 mins) detection of WSSV raw infected shrimp tissue samples.

The conventional analytical techniques available for WSSV detection are based on polymerase chain reaction (PCR), DNA microarray^7^,^8^ dot blot, western blot^9^, enzyme linked immunosorbent assay (ELISA)^10^,affinity immunosensor^11^,^12^, and antibody based microarray methods^13^. Meanwhile, sandwich immunoassay test kits were also developed recently^14^,^15^,^16^. Unfortunately, all these molecular techniques have their own limitations such as; less sensitivity and high detection limit (~10^3^ copies of its gene μL^−1^), complicated offline preparation procedures, involvement of carcinogenic chemicals like ethidium bromide (in polymerase chain reaction (PCR)), requirement for well-equipped laboratories (for DNA assays), trained technicians and time consuming measurements (For ELISA 2–3 days’ of time period required) and limited to qualitative assay (ELISA kit). Alternately, electrochemical techniques seem to be a promising for elegant immunosensing. It allows development of low cost miniaturize-microelectronics suitable for simple, quick detection of targeted analyte in raw real samples. Indeed, development of pathogen selective electrochemical immunosensor is a challenging research area. Previously, a couple of DNA based bio-electrochemical sensors were developed for the WSSV detection^17^,^18^. Zhang et al reported a thiol functional group modified ssDNA (probe) self-assembled monolayer micro-electromechanical system as a sensor electrode and ferricyanide as a transducer for hybridization detection of targeted ssDNA (WSSV)^17^. Kongpeth *et al* reported a anthraquinone-labeled pyrrolidinyl peptide nucleic acid (AQ-PNA) probe based immobilization-free detection of WSSV-ssDNA^18^. Note that, in the above DNA biosensors, several complicated and time consuming off-line preparation procedures including PCR or Loop mediated isothermal amplification (LAMP) amplification procedures have been used. To the best of our knowledge, an electrochemical immunosensor approach is never reported for the WSSV detection in the literature. A new electrochemical immunosensor, introduced in this work, showed sensitive analysis of WSSV in raw tissue samples (Fig. 1), which has been collected by 10 min homogenization and centrifugation of raw tissue samples with Tris EDTA buffer, unlike to the time-consuming PCR/LAMP based WSSV sensing approaches.

Graphene oxide (GO) has been frequently used as a matrix in electrochemical biosensors owing to its unique chemical structure and biocompatibility feature. For instance, silver nanoparticles/SiO_2_/graphene oxide hybrid modified glassy carbon electrode for the electrochemical immuno-sensensing of potent synthetic estrogenic hormones, Ethinylestradiol, silver nanoparticles-reduced graphene oxide-indium-tin-oxide (ITO) modified electrode for electrochemical immunosensing for carcino embryonic antigen^19^, gold nanoparticles-GO based electrochemical immunosensor for a tumor suppressor protein, p53^20^, and GO-chitosan-ferrocene-gold nanoparticle based electrochemical immunosensor for human carcinoembryonic antigen^21^. It is noteworthy that either gold or silver nanoparticles coupled secondary antibodies (Horseradish peroxidase (HRP) linked antibody, Ab2-HRP), have been frequently used in their electrochemical immunosensor assays. Unfortunately, gold and silver nanoparticles can itself interact with hydrogen peroxide even without HRP enzyme and hence can produce false positive value in the respective electrochemical immunosensors. In addition, dissolved oxygen will interfere very seriously at the H_2_O_2_ detection potential. In this work, gold or silver nanoparticle-free electrochemical immunosensor platform based on a GO-methylene blue (MB) dye modified glassy carbon electrode, designated as GCE/GO@MB, and has been introduced. This new electrochemical immunosensor showed highly sensitive and selective detection of WSSV without any false positive result and dissolved oxygen interference. As a proof of concept selective detection of WSSV in a couple of shrimp samples were demonstrated. Following are the merits of present sensing approach; (i) first report for the electrochemical immunosensing of WSSV, (ii) simple, sensitive and quick analysis of real sample, (iii) the lowest detection limit obtained in this work is the lowest value (1.36 × 10^−3^ copies μL^−1^) ever reported for the WSSV, (iv) use of raw tissue samples, unlike to the PCR/LAMP amplified samples in the conventional techniques, for real sample analysis and (iv) suitable for both qualitative and quantitative analyses.

## Result and Discussion

**Figure 1:**
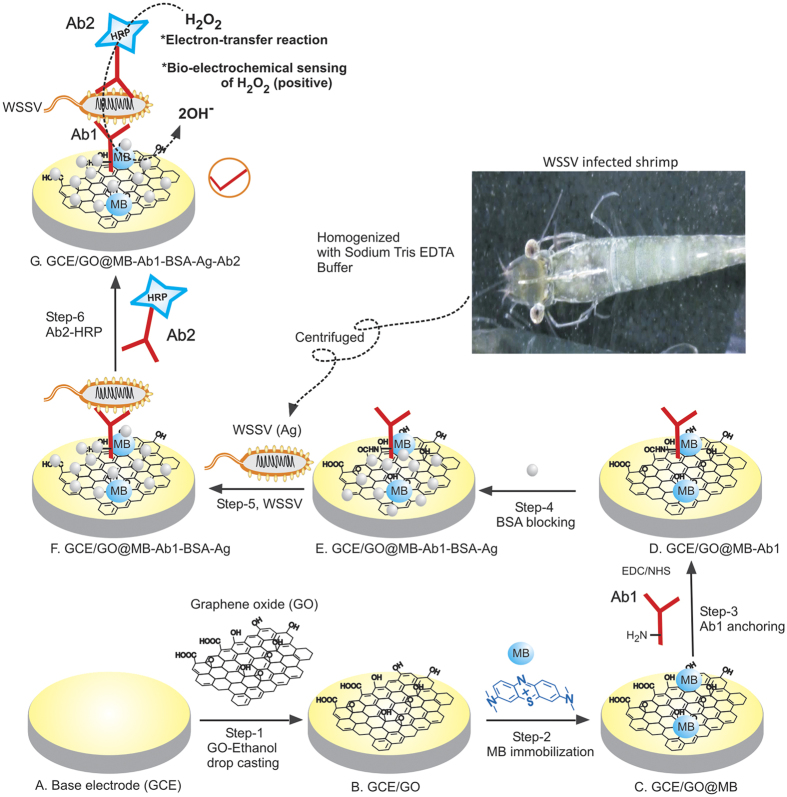
Illustration for the development of WSSV electrochemical immunosensor using bare GCE by sequential modification of GO (Step-1), MB (Step-2), Ab1 (Step-3), bovine Serum Albumin blocking (Step-4), WSSV target Ag (vp28) (Step-5) and Ab2-HRP (Step-6) and its mechanism for the bio-electrocatalytic H_2_O_2_ reduction reaction. Inset is a photograph of WSSV infects shrimp.

**Figure 2:**
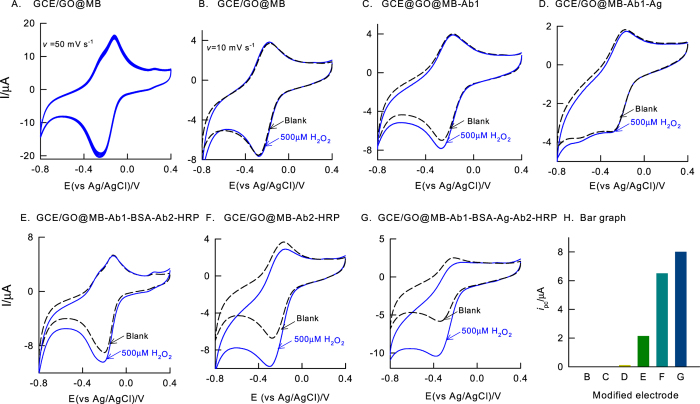
CV responses of (**A**) GCE/GO@MB, (**B**) GCE/GO@MB, (**C**) GCE/GO@MB-Ab1 (**D**) GCE/GO@MB-Ab1-Ag, (**E**) GCE/GO@MB-Ab1-BSA-Ab2-HRP, (**F**)GCE/GO@MB/ Ab2-HRP, (**G**) GCE/GO@MB-Ab1-BSA-Ag-Ab2-HRP without (Blank) and with 500 μM of H_2_O_2_ in pH 7 PBS at *v* = 10 mVs^−1^. (**H**) A plot of *i*_pc_ of H_2_O_2_ vs different modified electrodes.

The fabrication of the immunosensor is clearly shown in Fig. 1, which includes modification of GO, methylene blue, primary antibody (Ab1) modification, BSA blocking, WSSV antigen (Ag) and HRP labelled secondary antibody (Ab2 or Ab2-HRP) for virus detection. Initial experiments were focused on the optimization of the electrochemical immunosensor parameters. Figure 2(A) is a ten continuous CV response of GCE/GO@MB, prepared by immersing GO modified GCE, GCE/GO in 5 mg MB dissolved 500 μL ethanol solution for 5 min, showed a well-defined redox anodic and cathodic peak at an equilibrium potential (E_1/2_ = E_pa_ + E_pc_/2, where E_pa_ and E_pc_ are anodic and cathodic peak currents) value found to be −0.3 ± 0.005 V vs. Ag/AgCl in pH 7 PBS. The observed peak potential difference, ΔE_p_ = 160 mV suggesting a quasi-reversible behaviour of MB immobilised electrode system. When the electrode was gently washed with distilled water and CV was performed again, there was no alteration in peak current or peak potential noticed. Calculated relative standard deviation value for anodic peak current is 2.1%. Based on the experimental results and literature data^23^, it can be postulated that MB dye is adsorbed strongly on the underlying GO surface and showed the redox activity. It is likely that π-π interaction between the aromatic portion of MB and sp^2^ carbon of GO is responsible factor for the stability of the modified electrode. This point onwards the modified electrode is designated as GCE/GO@MB.

**Figure 3:**
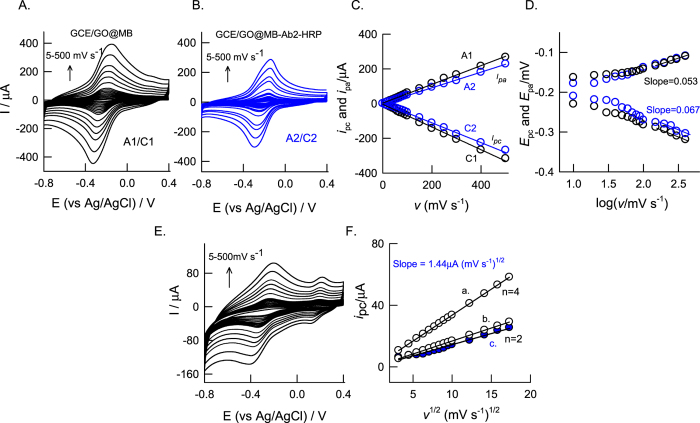
CV responses of GCE/GO@MB (**A**) and GCE/GO@MB-Ab2-HRP (**B**) at different scan rates (5–500 mV s^−1^) in pH7 phosphate buffer solution. Plots of (**C**) anodic (*i*_pa_) and cathodic (*i*_pc_) peak currents vs scan rate and (**D**) anodic (E_pa_) and cathodic (E_pc_) peak potentials vs log (scan rate) for the GCE/GO@MB (A1/C1) and GCE/GO@MB-Ab2-HRP (A2/C2). CV response of (**E**) GCE/GO@MB-Ab2-HRP in 500 μM H_2_O_2_ dissolved pH 7 PBS at different scan rate (5–500 mV s^−1^) and (**F**) Plot of *i*_pc_ of H_2_O_2_ vs square of scan rate for the GCE/GO@MB-Ab2-HRP.

Suitability of the GCE/GO@MB for electrochemical immunosensor was tested by performing hydrogen peroxide interaction in pH7 PBS as in Fig. 2(B). The GCE/GO@MB system failed to show any alteration in the peak current and potential without and with 500 μM H_2_O_2_ indicating absence of any electro-catalytic activity of the underlying electrode. It is a clear advantage of using this surface-confined redox system further for H_2_O_2_ biosensor application. As control experiments, individual and combinations of antigen (WSSV-vp28), Ab1 and Ab2 (i.e, Ab2-HRP) systems modified on GCE/GO@MB prepared as per the procedure given in Fig. 1, were subjected to H_2_O_2_ electrochemical reduction reaction. As can be seen in the Fig. 2(C,D and E), Ab1, Ag-Ab1 and Ab1-Ab2 modified GCE/GO@MB systems showed either nil or very feeble redox peaks due to unavailability of HRP (Ab1) and improper binding of antibody systems (Ab1-Ab2) on the working electrode surface respectively. In further, Ab2 modified electrode, GCE/GO@MB-Ab2 was tested for the activity Fig. 2(F). Interestingly, a well-defined electro-catalytic reduction response for H_2_O_2_ at a reduction peak potential, −0.35 V vs Ag/AgCl, where the MB redox peak exist, was noticed in Fig. 2(G). Effect of scan rate on the CV response of the modified electrodes, GCE/GO@MB and GCE/GO@MB-Ab2 showed a systematic increase in both anodic and cathodic peak currents (Fig. 3(A and B)). A plot of base-line corrected peak currents, *i*_pa_ and *i*_pc_ against scan rate (*v*) for both of the modified electrodes showed a linear line starting from origin indicating surface-confined electron-transfer mechanism of the systems (Fig. 3(C)). This observation highlights elegant immobilization and effective electron-transfer shuttling of Ag and Ab2-HRP protein with MB.

The surface concentration of MB-electro active species (*Γ*_*MB*_, mol cm^−2^) in GCE/GO@MB and GCE/GO@MB-Ab2 can be calculated from the slope of the peak currents vs. scan rate Fig. 3(C). For a reversible reaction, the peak current is given by


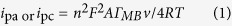


where *n* is the number of electrons transferred, *F* is the faraday constant (96500), *A* is the geometrical area of the electrode, *v* is the potential scan rate. From the above equation, the calculated surface concentration of MB was estimated to 9.84 × 10^−10^ mol cm^−2^ and 8.41 × 10^−10^ mol cm^−2^ for GCE/GO@MB and GCE/GO@MB-Ab2 respectively. Electro-kinetic parameter such as transfer coefficient (α) and rate constant (*k*_s_) values for surface-confined MB were calculated based on scan rate data and Laviron^24^ electrokinetic model by plotting the variation of anodic and cathodic peak potentials with logarithm of scan rate Fig. 3(D). It obeys the procedure of Laviron by indicating the *E*_pa_ or *E*_pc_ values are proportional to logarithm of scan rate for values higher than 0.2 V s^−1^. The slope of the plots can provide the kinetic parameters α_c_ and α_a_ (cathodic and anodic transfer coefficients). The slope of the linear segments are equal to -2.303RT/αnF and 2.303RT/(1-α)nF for the cathodic and anodic peaks^25^ respectively, and the calculated values for α in pH 7 PBS for with and without secondary antibody are 0.53 and 0.45 respectively.


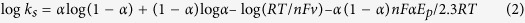


Based on the equation (2), calculated *k*_s_ values for GCE/GO@MB and GCE/GO@MB-Ab2 in PBS are 1.65 ± 0.5 s^−1^ and 1.04 ± 0.5 s^−1^ respectively. A slightly lowered surface excess and *k*_s_ values observed with the GO@MB-Ab2 modified electrode than that of the unmodified electrode is due presence of the electro-inactive protein species (Ab2) in the modified electrode.

Electrocatalytic H_2_O_2_ reduction rate constant of GCE/GO@MB-Ab2, *k*_chem_, can be calculated with help of the expression for the catalytic current (*i*_pc_) given by Andrieux and Saveant for a catalytic reaction in the case of slow scan rate and large *k*_chem_^26^;


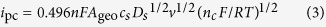


It has been shown that in the case of fast scan rates and low *k*_chem_ the values of the “constant” in equation are lower than 0.496^27^. As per Fig. 1 in the published paper by Andrieux and Saveant study,^26^ where a working curve for the “constant” 0.496 was given as a function of


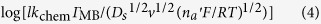


For GO@MB-Ab2 system with 500 μM H_2_O_2_. the average value of this coefficient is found to be 0.22 (calculated by referring the working curves based on the data calculated *k*_chem_, in the scan range 100 to 500 mV s^−1^). Thus *k*_chem_ was calculated as 1.1 × 10^3^ mole^−1^ dm^3^ s^−1^.

In further, number of electrons involved in the H_2_O_2_ electrochemical reduction reaction (*n*) was theoretically calculated using *Randles Sevick* equation assuming quasi-reversible behaviour of the reduction system as,


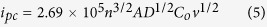


where, *i*_pc_ is reduction peak current, *A* is the electrode area (0.0707 cm^−2^), *D* is the diffusion coefficient of methylene blue (1.71 × 10^−5^ cm^2^ s^−1^)^28^ and *C*_o_ is the concentration of analyte used (500 μM). By keeping *n* = 2 or *n* = 4, respective *i*_pc_ values were back calculated from the eqn (5) and plotted against *v*^1/2^ as in Fig. 3(F), curve a (n = 2) and b (n = 4). In addition, the experimental *i*_pc_ value also plotted in the same graph (Fig. 3F, curve c). It is obvious that the *n* = 2 theoretically simulated curve is fitting very well with the experimental observation ascribing involvement of *n* = 2 in the overall H_2_O_2_ reduction reaction. Meanwhile, a pre-peak at 0.2 V vs Ag/AgCl was specifically noticed at scan rate on optimal electrode as in Fig. 3E, which is due MB at energetically different GO sites possibly on graphitic and non-graphitic (oxygen containing surface edge) sites. Indeed, MB immobilized on graphitic sites of GO shows predominant electrocatalytic feature and thus it is taken for further analysis.

**Figure 4:**
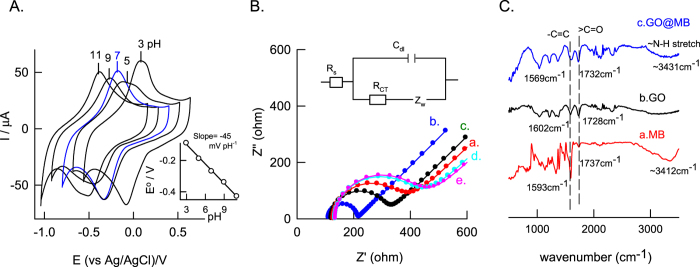
(**A**) Effect of pH on CV of GCE/GO@MB at *v* = 50 mV s^−1^ and inset plot is E° vs pH, (**B**) Electrochemical impedance responses obtained with the bare GO (a), GO@MB (b), GO@MB-Ab1 (c), GO@MB-Ab1-BSA-Ag (d), GO@MB-Ab1-BSA-Ag-Ab2 (e) in 0.1 M KCl solution containing 5 mM each of [Fe(CN)_6_]^3−^ and [Fe(CN)_6_]^4−^ redox probe at an applied potential 0.3 V vs Ag/AgCl. The concentration of WSSV antigen is 1 μg μL^−1^. The impedance spectra were recorded in a range, 0.1 Hz–100 KHz. (**C**) Comparative FTIR/KBr responses of MB (a), GO (b) and GO@MB (c).

Inorder to exemplify the effect of pH, GCE/GO@MB was subjected to CV at various pH solutions *viz*. pH 3,5,7,9 and 11 (Fig. 4). The observed results shows that the formal potential value shifted to negative direction with increase in pH (Fig. 4A). A plot between formal potential (E°/V) vs pH is found to be linear with a slope and regression coefficient values of −45 ± 3 mV pH^−1^. The obtained slope value is less than that the theoretical slope value of −59 mV pH^−1^ for equal number of proton and electron in the electrochemical reaction suggesting that Non-Nersntian behaviour with involvement of 2 H^+^/3e^−^ in the electrochemical reaction mechanism.

Feasibility of the GCE/GO@MB system was tested by performing electrochemical immunosensing of WSSV via electrochemical ELISA in presence of 500 μM H_2_O_2_. For the preparation of the electrochemical immunosensor, following order was followed, Ab1 →  → BSA → Ag → Ab2-HRP as in the Fig. 1. CV response of the sandwich electrochemical ELISA system without and with 500 μM H_2_O_2_ in pH7 PBS at a scan rate 10 mV s^−1^ was shown in Fig. 2(G). A clean H_2_O_2_ reduction signal was observed with the electrochemical immuno-system modified electrode. Three repeated experiments of electrochemical sensing of WSSV-vp28 yielded a RSD 4.7%. This observation authenticates the facile immunochemical reaction on the GCE/GO@MB surface for further electrochemical quantification.

Part of the modified electrodes have been characterized using electrochemcial impedance spectroscopy (EIS) and FTIR techniques. EIS were employed to study the interfacial properties of the electrode after each step of modification. Figure 4B shows the impedance response in a step by step modification of the immunochemical sensor at an applied potential, 0.3 V vs Ag/AgCl with 5 mM each of [Fe(CN)_6_]^3−^ and [Fe(CN)_6_]^4−^ in 0.1 M KCl solution (as per Fig. 1). A semicircle coupled linear line like responses were uniformly observed due to charge transfer reaction (R_CT_) and analyte diffusion processes respectively with all the test systems. The respective R_CT_ values were calculated using Randles equivalent circuit, in which, a solution resistance (R_s_) and double layer capacitance (C_dl_) are arranged in parallel with R_CT_ and Warburg resistance (Z_W_) (Fig. 4B inset)^29^. Calculated R_CT_ values of modified electrodes prepared by stepwise procedure are; GO@MB = 103 ohm, GO = 188 ohm, GO@MB-AB = 229 ohm, GO@MB- Ab1-Ag = 272 ohm, GO@MB- Ab1-Ag-Ab2 = 284 ohm. It is obvious that the R_CT_ values increased regularly in the stepwise procedure. This observation might be due to dielectric and insulating features of the antibody and antigen that have been modified on the electrode surface. Although these results demonstrate feasibility of development EIS based electrochemical immunosensor for WSSV, with respect to selectivity and reliability bio-electrocatalytic sensor based electrochemical immunoassay system is superior than that of the impedimetric sensor.

**Figure 5:**
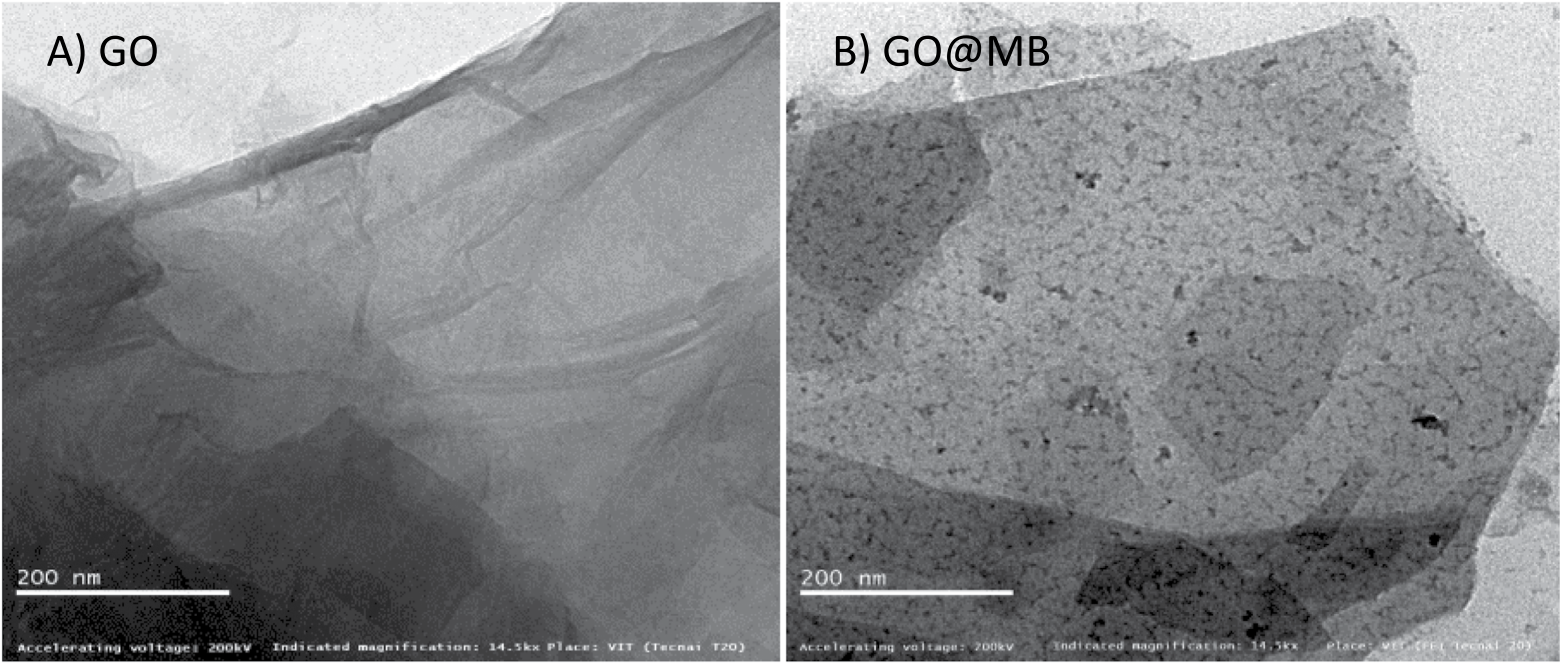
Transmission Electron Microscope images of GO (**A**) and MB surface confined GO (**B**).

To further characterize the immobilisation of methylene blue on graphene oxide, comparative FTIR spectroscopy was carried out with GO@MB, GO and MB as in Fig. 4C. A vibration signal due to C = C (1732 cm^−1^, 1728 cm^−1^, 1737 cm^−1^) and > C = O (1569 cm^−1^ and 1602 cm^−1^) were qualitatively noticed with all the samples (No > C = O signal with MB); but with a shift in the wavenumbers. In addition, specific vibrational band at 3412 cm^−1^ corresponds to the N-H stretching of MB was also noticed with GO@MB (3431 cm^−1^) ascribing MB immobilization and its GO interaction features. The morphological structure of GO and GO@MB was investigated using TEM Fig. 5(A and B). A transparent sheet like structure can be seen clearly with the GO as that of previously reported literature^22^. For MB modified GO case, large number of black spots of average size ~10 nm on the GO sheets was observed. This observation can be taken as a proof for the immobilization of MB on GO.

**Figure 6:**
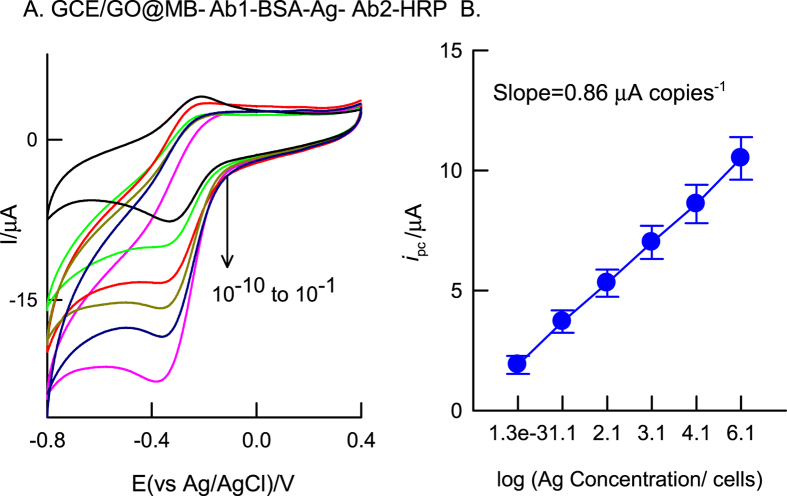
CV responses of GCE/GO@MB-Ab1-BSA-Ag-Ab2-HRP, prepared with different dilution of antigen (10^−1^ to 10^−10^), in 500 μM H_2_O_2_ containing pH 7 PBS at *v* = 10 mV s^−1^.

Next, calibration plot for electrochemical immunosensing of WSSV was constructed by subjecting different concentration of the standard virus (vp28 protein) discreetly on the modified electrode as GCE/GO@MB-Ab1-BSA-Ag-Ab2 and subjected to CV experiment with fixed H_2_O_2_ concentration (500 μM) in pH 7 PBS. Figure 6 is the typical calibration response for the virus obtained from different dilutions (10^−1^ to 10^−10^) of the stock WSSV, 1.37 × 10^7^ copies μL^−1^. The sensor showed regular variation in the H_2_O_2_ reduction current with respect to dilution. Note that the detection range (1.37 × 10^−3^–1.37 × 10^7^ copies μL^−1^) and low-detection concentration (1.37 × 10^−3^ copies μL^−1^) obtained in this work are much better than the previous reported procedures like label-free affinity immunosensors (1.6 × 10^3–1.6^ × 10^6^ copies μL^−1^ and 1.6 × 10^1^–1.6 × 10^6^ copies μL^−1^) and colorimetric ELISA (1.6 × 10^3^–1.6 × 10^7^ copies μL^−1^)^11^,^12^,^30^.

**Figure 7:**
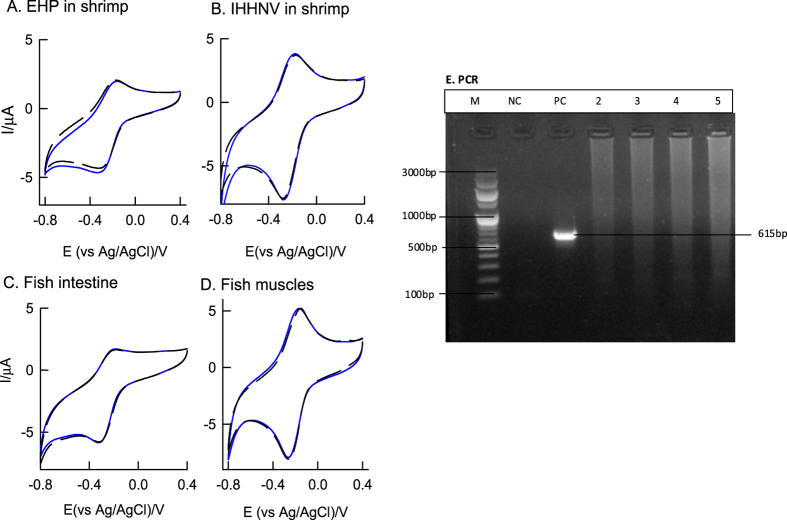
CV responses of different antigens modified GCE/GO@MB-Ab1-BSA-Ag-Ab2-HRP without and with 500 μM H_2_O_2_ containing pH 7 PBS at v = 10 mV s^−1^; (**A**) *Enterocytozoon Hepatopenaei*, (**B**) Infectious *Hypodermal* and *Haematapoietic Necrosis Virus*, (**C**) Fish intestine and (**D**) Fish Muscle. (**E**) Agarose gel showing PCR amplification of WSSV vp28 gene. M-100 bp DNA Marker, NC-Negative Control, PC-positive control-WSSV (white Spot Syndrome Virus), 2-IHHNV (Infectious Hypodermal and Haematopoietic necrosis Virus) DNA, 3-EHP (*Enterocytozoon Hepatopenaei*) DNA, 4-*vibrio parahaemolyticus* infected Fish intestine, and 5-*vibrio harveyi* infected fish muscle samples.

Electrochemical immunosensor specificity is major concern in the real sample analysis. Figure 7(A–D) is CV response of the GCE/GO@MB for various cross-reactivity samples based on different aquatic pathogens such as EHP, IHHNV, *Vibrio parahaemolyticus* and *Vibrio harveyi.* For the preparation of different aquatic pathogen modified GCE/GO@MB electrodes, procedure mentioned in Fig. 1 and Fig. 2G was adopted. Interestingly, these immunosensor systems failed to show any signal for H_2_O_2_ reduction current, unlike to the WSSV-vp28, postulating the selectiveness of the present sensor for the WSSV. The specificity study was cross-confirmed with discreet PCR as well. Fig. 7(E) is the typical PCR analysis result of WSSV-infected positive DNA (PC, vp28) and healthy animals-negative DNA (NC) samples. The symbol “M” in the PCR photograph is the standard commercial 100 base pair DNA ladder. As can be seen, there were no target specific bands observed with the cross-reactivity samples when compared with the controls.

**Figure 8:**
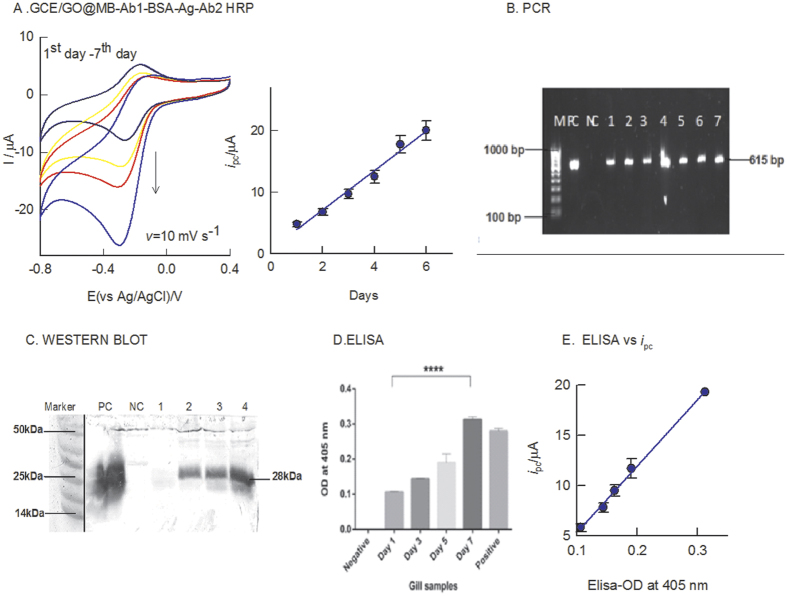
CV response of different days WSSV infected gill tissues (fresh water crabs; *Paratelphusa hydrodomous*) modified GCE/GO@MB-Ab1-BSA-Ag-Ab2-HRP with 500 μM H_2_O_2_ containing pH 7 PBS at *v* = 10 mV s^−1^. Inset is a plot of i_pc_ of H_2_O_2_ reduction reaction vs different day samples. (**B**) PCR of the different day samples analysed. M-100 bp marker, PC- positive control, NC-negative control, 1–7 lanes denote day-1 to day-7 of gill tissue samples. (**C**) Western Blot of different days’ gill tissue samples. PC-positive control, NC-negative control, 1 = 1st day gill tissue, 2 = 3^rd^ day gill tissue, 3 = 5^th^ day gill tissue & 4 = 7^th^ day gill tissue. (**D**) ELISA of time course study gill sample. (**E**) Plot of the optical density of ELISA measured at 405 nm vs peak current (*i*_pc_) of the different day sample.

As a proof of concept and usefulness of this protocol, selective detection of WSSV in raw gill tissues during the course of infection until it reaches moribund stage that usually take about 7 days were examined. Each sample is subjected to triplicate measurements. For convenience, 1^st^ data result was presented in this work. The electrochemical immunosensor preparation procedure follows similar to Fig. 1. Figure 8(A) is a CV response of progression of the WSSV in the infected gill tissue from day 1 to day 7. In parallel, investigations were also carried out using PCR, western blot and conventional ELISA techniques along with positive and negative controls as in Fig. 8(B) to (D) respectively. As seen in Fig. 8(B), the PCR analyses gave specific bands for the 1–7 day samples relating to the qualitative information of the pathogen. There is no significant variation in the band intensities of PCR for the different time duration samples. Similarly, western blot Fig. 8(C) and ELISA Fig. 8(D) analyses results also gave signals only from the day 3 of the post infection. Interestingly, the electrochemical immunosensor results showed specific current signals for all the time duration samples. As can be seen in the Fig. 8(A) and (E), the current signals were increasing proportionately with increase in the post infection time. A plot of immunosensing signal vs post infection time (1–7 day) showed a linear line with slope value of 3.2 μA per day. In addition, a plot of electrochemical signal vs ELISA@ 405 nm OD was found to be linear Fig. 8(E), confirming suitability and reliability of the electrochemical sensor for further routine analysis.

## Conclusion

A methylene blue dye immobilized graphene oxide modified glassy carbon electrode was constructed as an electrochemical immunosensor platform for the quick and raw sample analysis of white spot syndrome virus in *penaeid* shrimp and crab samples. The immunosensor system showed highly selective bio-electrocatalytic signal to hydrogen peroxide utilizing the HRP linked secondary antibody with methylene blue on the modified electrode surface. This new electrochemical immunosensor system did not show any false positive results in the absence of secondary antibody and tolerable to dissolved oxygen interference. Tested cross-specificity experiments with EHP, IHHNV, *Vibrio parahaemolyticus* and *Vibrio harveyi* pathogens failed to show any signal to H_2_O_2_ reduction reaction. Constructed calibration plot was linear in WSSV-vp28 copies concentration window 1.37 × 10^7^ to 1.37 × 10^−2^ copies μL^−1^ with a regression coefficient and sensitivity values of 0.99 and 0.86 μA copies^−1^ respectively. Applicability of the electrochemical immunosensor was demonstrated by analyzing the course of WSSV infection until moribund stage. Obtained results were compared with the PCR, Western blot and conventional ELISA analyses. It is obvious that both qualitative and quantitative analyses of the WSSV were achieved in this new electrochemical immunosensor platform, unlike to the qualitative results with existing conventional techniques.

## Experimental section

### Chemicals and Reagents

Graphene oxide (>80% carbon basis flake size-0.5–2.0 μm, thickness- 0.6–1.2 nm), methylene blue, 3-dimethyl aminopropylcarbodiimide/*N*-hydroxysuccinimide (EDC/NHS), bovine serum albumin, ethidium bromide, 1% agarose gel, 2,2′-Azinobis (3-Ethylbenzthiazoline-6-Sulfonate) (ABTS), sodium dodecyl sulphate (SDS), were all purchased from Sigma-Aldrich (USA).TMB (3,3′, 5,5′-tetramethylbenzidine) blotting developing substrate solution was obtained from Thermo Fisher (India). Glycerol, 2-mercaptoethanol, bromophenol blue, TrisHCl, ethylene diamine tetra acetic acid (EDTA), guanidine hydrochloride, sodium acetate, tween 20 solutions were obtained from SRL chemicals, India. The WSSV gene, vp28 was collected from Xcelris, India. 2× master mix for standard PCR reaction setup and secondary antibody (Ab2-HRP conjugate) were obtained from Genei, Bangalore, India. Other chemicals, which used were all ACS-certified reagent grade and used without purification. Aqueous solutions were prepared using deionized and alkaline KMnO_4_ distilled water. The supporting electrolyte pH 7 phosphate buffer solution (PBS) of ionic strength = 0.1 mol L^−1^ was used throughout the work. ***Caution!** Because ethidium bromide is carcinogenic, proper care must be taken during handling*.

### Instrumentation

DNA quantification was done using UV-vis spectrophotometer (ELICO, India). PCR amplification of WSSV structural gene, vp28 was performed using Eppendorf thermal cycler, Belgium. For ELISA analysis, LARK, California instrument was used. SDS-PAGE gel was performed using MEDOX SDS, India apparatus. A semi-dry blot (Bench top, India) apparatus was used for the membrane transfer in western blotting. Voltametric measurements were carried out with a CHI440B instrument (USA). The three electrode system consists of glassy carbon electrode (GCE) or its chemically modified system (0.0707 cm^2^) as a working electrode, Ag/AgCl (3 M KCl) as a reference electrode and platinum wire as a counter electrode. Transmission electron microscope (TEM) was done using a Technai, G2 20 Twin FEI instrument (Czech Republic). Impedance spectroscopy studies were carried out with Metrohm Autolab^®^, Netherlands. FTIR spectroscopic measurements were performed with JASCO 4100 (Japan) spectrophotometer using Kbr method.

### Molecular technique experiments

Fresh water crabs (*Paratelphusahydrodomous)* were collected from the rice field near VIT University. The crabs weighed around 10–15 grams were carried to laboratory and acclimatized in 5 L reverse osmosis (RO) treated water tanks. Prior to the experiment, the crab samples were stored in room temperature and fed with egg-white twice daily. Periodically, water tanks were cleaned and the water was changed. Preparation of viral inoculum was followed as per the reported procedure^31^. The animals were divided into two groups, one as control and the other infected. Each group contains six animals and the experiment was carried out in triplicates. Progress of the infection was monitored twice a periodically.

The moribund animals from infected group and the healthy animals from uninfected groups were sacrificed and gills and muscle tissues were dissected in accordance to the guideline given in the previously recommended procedure^32^. Guanidine hydrochloride method was adopted for DNA extraction from dissected tissues^33^. DNA quantification was done by measuring the absorbance at 260 nm and 280 nm in UV-vis spectrophotometer. The amplification of WSSV structural gene, vp28 was done using 20 μL standard PCR reaction contains 10 μL of 2 × master mix, 5 pmol vp28 primer (Forward- ATG GAT CTT TCT TTC AC and Reverse- TTA CTC GGT CTC AGT GC), 3 μl PCR grade water and 1 μL template DNA. The cycling condition initially starts with denaturation 95 °C for 5 minutes then 35 cycles of denaturation at 95 °C for 30 seconds followed by annealing 50 °C for a minute and extension at 72 °C (1 minute) and final extension at 72 °C for 10 minutes. The PCR product was checked in 1% agarose gel with ethidium bromide being used as a staining agent and the results were documented. The total protein from tissue homogenate was estimated by using Bradford’s protein estimation procedure^34^. Proteins were mixed with Laemmli sample buffer for SDS-PAGE analyses^35^.WSSV virions from purified vp28 protein served as a positive control, while healthy gill tissue was included as a negative control. Western blot and ELISA experiments were performed as per the literature procedures^36^,^37^. The anti-mouse vp28 polyclonal antibodies at a dilution of 1: 10,000 were used as primary antibody (raised in rabbit) and HRP linked primary antibody at a dilution of 1: 10,000 were used as secondary antibody for detection of vp28 (goat raised anti-rabbit). In the ELISA procedure, 96 well micro-liter plates coated with 100 ng well^−1^ incubated overnight at 4 °C of *vp*28 antigen was used. For colouring reaction, ABTS (2,2′-azinobis-(3-ethylbenzothiazoline-6-sulfonic acid)) was used and results were measured at 405 nm.

The specificity of *vp*28 gene was analysed parallelly using electrochemical immunosensor and PCR. Various non-specific pathogenic samples including Enterocytozoon Hepatopenaei (EHP) of shrimp, Infectious Hypodermal and Haematapoietic Necrosis Virus (IHHNV) of shrimp*, Vibrio Parahaemolyticus* infected fish intestine and *vibrio harveyi* infected fish muscle were used.

WSSV positive tissue was homogenized using mortar and particle with 10% suspension of sodium Tris EDTA buffer (NTE). The clear homogenate centrifuged at 5000 rpm for 5 minutes was directly subjected to electrochemical immunosensing experiment. A [WSSV-vp28] = 1.37 × 10^7^ copies μL^−1^, quantified using PCR, was used as a stock solution. Serial dilutions, 10^−1^ to 10^−10^ were prepared from the clear homogenate (stock) with NTE buffer. Base-line corrected hydrogen peroxide (500 μM) reduction peak current was taken as analytical parameter in the electrochemical immunosensing.

### Fabrication of GCE/GO@MB modified electrochemical immunosensor

Glassy carbon electrode (GCE) was cleaned and polished using alumina powder. Initially, a 5 μL of GO from the GO-ethanol stock solution (5 mL) was casted on a clean GCE (GCE/GO) and dried in air for 5 ± 1 mins at room temperature (27 ± 2 °C) (step-1). Methylene blue dye was immobilized on GCE/GO by immersing the working electrode in 5 mg MB dissolved 500 μL ethanol solution for 5 mins (step-2). Prepared electrode was pre-treated by performing continuous CV in pH 7 PBS at scan rate (*v*) = 50 mV s^−1^ for twenty cycles in pH 7 PBS. In step-3, primary antibody was modified on the GCE/GO@MB. Prior to the experiment, GO surface functional group (-COOH and NH_2_) was activated by treating with 1:1 ratio of EDC-NHS (10 mg mL^−1^) solution for 5 mins. Subsequently the electrode was drop-casted with 1 μL of primary antibody (Ab1) (1 mg mL^−1^) and dried for 5 mins in room temperature (27 ± 2 °C). Step-4, the electrode was treated with 1% (w/v) (1 mg mL^−1^) bovine serum albumin (BSA) in 0.1 M PBS, pH 7 and dried at room temperature for 5 mins to block the non-specific binding sites and then rinsed with milli-Q water to remove unbounded BSA. Step-5, WSSV incubation was performed by coating the above modified electrode with 1 μL of antigen (WSSV inoculum) and dried for 5 mins in room temperature. Finally, in 6^th^ step, the modified electrode was overlaid with 1 μL of HRP labelled secondary antibody (Ab2-HRP) (1 mg mL^−1^), dried for 5 mins in room temperature and washed with water. Then, GCE/GO@MB-Ab1-Ag-BSA-Ab2-HRP was subjected to CV study with 500 μM H_2_O_2_ dissolved pH 7 PBS at v = 10 mV s^−1^. By this method, we are able to modify the immunosensor to sense the presence or absence of WSSV in the homogenized animal tissue sample within 35 ± 5 min time.

## Additional Information

**How to cite this article**: Natarajan, A. *et al*. An Elegant Analysis of *White Spot Syndrome Virus* Using a Graphene Oxide/Methylene Blue based Electrochemical Immunosensor Platform. *Sci. Rep.* **7**, 46169; doi: 10.1038/srep46169 (2017).

**Publisher's note:** Springer Nature remains neutral with regard to jurisdictional claims in published maps and institutional affiliations.
